# A Control Method for Optimizing the Spectral Ratio Characteristics of LED Lighting to Provide Color Rendering Performance Comparable to Natural Light

**DOI:** 10.3390/s25247453

**Published:** 2025-12-07

**Authors:** Seung-Teak Oh, Ji-Young Lee, Jae-Hyun Lim

**Affiliations:** 1 Smart Natural Space Research Center, Kongju National University, Cheonan 31080, Republic of Korea; ost73@kongju.ac.kr; 2 Department of Computer Science & Engineering, Kongju National University, Cheonan 31080, Republic of Korea; easy1814@smail.kongju.ac.kr

**Keywords:** natural light, LED, lighting, CRI, spectral power distribution (SPD), optimization of spectral ratio

## Abstract

Light-emitting diode or LED lighting often faces challenges with color rendering due to its unique spectral characteristics compared to natural light. While efforts to enhance the color rendering index (CRI) have typically focused on improving light source elements, there has been less attention on optimizing the software aspects, such as the combination of light sources and spectral composition. Notably, there has been no efficient method proposed specifically for enhancing R9 and R12, which are critical for improving overall color rendering in LED lighting. This paper presents an optimization control method based on the spectral ratios of LED lighting to achieve color rendering similar to natural light. By analyzing the wavelength characteristics of both natural and artificial light, a high CRI light was realized through reinforcement of deficient wavelength bands. Experimental results showed an average CRI of 97, with R9 and R12 values around 93 and 98, respectively, demonstrating that LED technology can achieve color renderings comparable to natural light.

## 1. Introduction

Color rendering performance is a critical evaluation factor for light quality [1]. Recently, lighting technology that can show the color of an object as natural light has been highlighted. Natural light provides the best light for humans to recognize objects and distinguish colors [2]. On the other hand, artificial lighting, especially the most commonly used LED lighting, has less color rendering performance than natural light [3,4]. These differences arise from the differences in the spectral characteristics of natural light and LED lighting [5]. Natural light displays the entire spectrum of the visible light wavelength band (380–780 nm). In contrast, general LED lighting often exhibits discontinuous spectral characteristics, resulting in a loss of clarity in specific colors [5,6]. The most popular color rendering performance indicator for general lighting is the Color Rendering Index (CRI), which evaluates how accurately the color of an object is rendered compared to natural light [7]. Earlier, only some colors of intermediate tone (R1 to R8) were targeted [8]. Recently, there have been increasing cases of applying special color rendering indices (R1 to R15) together to enable the evaluation of a broader range of colors, such as high saturation [9,10]. R9 and R12 are two key aspects of rendering that tend to be the weakest in general LED lighting, posing significant challenges in the lighting technology field [11]. The values of R9 and R12 decrease when there is a deficiency in spectral components above 600 nm and around 490 nm. When these values are low, it becomes difficult to accurately reproduce vibrant colors, particularly solid reds and blues, found in items like skin tones, fruit, clothing, the sky, and the ocean [12,13]. General LED lights may have low R9 and R12 values due to the characteristics of the SPD (Spectral Power Distribution), which include a peak wavelength near 450 nm and a cyan gap in the 470–520 nm band [14,15]. Efforts are ongoing to address this issue. Although it is theoretically possible to reproduce the spectrum of natural light as it is, it is technically challenging. Efforts are underway to achieve a wide and even spectral characteristic of the visible light wavelength band, as similar as possible to natural light [16]. For high color rendering performance lighting, the concept of “full visible spectrum lighting” has been proposed, and design methods for various phosphor combinations and packages have been studied [17,18]. High spectral uniformity was pursued by mixing and applying LED chips with different wavelength characteristics [19], and the spectrum of natural light was simulated by using an LED light source developed through the proposed powder sedimentation packaging method [20]. In addition, a spectral loss simulation was attempted to derive the correlation between specific color rendering characteristics, such as R9 and R12, and each spectral band [12,13]. A technology that provides light with high CRI under the daily changing natural light conditions was also introduced [21,22]. However, most research has focused on developing LED light sources with high CRI by combining phosphors or using multiple light sources, and there have been few efforts to achieve natural color rendering through software. Furthermore, even when high CRI was achieved, many studies reported results only under specific color temperature conditions. Although natural light was the intended focus, few studies have met the CRI performance standards, including R9 and R12, under the color-temperature conditions of daily natural light changes or have effectively analyzed and applied the characteristics of actual natural light. To achieve color rendering performance comparable to natural light, it is necessary to examine the SPD of actual natural light and develop an efficient control method to improve R9 and R2, which are weak in general LED lighting but have received little research attention.

This paper proposes a control method for high CRI natural light LED lighting that optimizes and reflects the spectral ratio characteristics of natural light. First, the seasonal/time-specific spectral characteristics of actual natural and artificial lights (three types of LED lights) were compared and analyzed. The spectral characteristics of each LED light were calculated for each wavelength band, and the most vulnerable wavelength band compared to natural light was explored. In addition, by performing a simulation with an SPD combination for each LED light source, a plan to reinforce and optimize the ratio of the deficient wavelength band was derived and then applied to realize control of the natural light LED lighting. In this study, the aim was to present a software method for applying the spectral characteristics of actual natural light to artificial lighting. Additionally, it introduced a lighting control technology designed to maintain a high CRI even as the color temperature of natural light changes.

## 2. Analysis of Natural and Artificial Light Characteristics for Spectral Ratio Optimization

**Figure 1:**
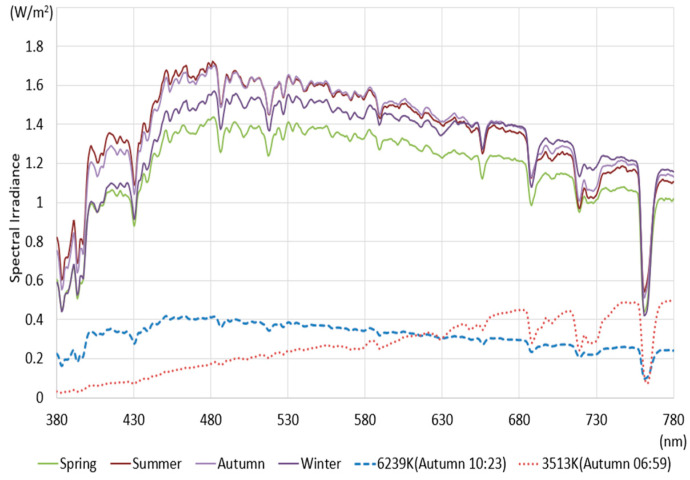
SPD of natural light (1 bright day for each season).

Natural light has a uniform spectral characteristic across the entire visible light wavelength band from 380 to 780 nm and provides a reference light for color rendering evaluation [6]. Therefore, to provide high color reproducibility at the level of natural light through artificial light, the differences between natural light and artificial light must be compared and analyzed. The color rendering performance can be improved by reflecting the spectral characteristics of natural light into artificial light. In this study, to confirm the difference between natural and artificial lights, the light characteristics of natural and artificial lights were measured, calculated, and analyzed. The characteristics of each light source were measured using a spectroradiometer (CAS 140CT, Instruments, Munich, Germany). Natural light was measured using a solar tracker and a constant temperature enclosure facility on the rooftop of a building at K University, located at latitude 36.851 and longitude 127.151. Measurements of natural light were conducted for approximately four years from 2021. One bright day per season was extracted from the collected results. Artificial light was measured directly from the ceiling using a lighting cabinet installed in a room where external light was blocked. The experimental lighting was selected as artificial light (LED lighting) manufactured by domestic company K. The selected artificial lights included three types: general office lighting (Light0), color temperature tunable lighting (Light1), which is capable of providing a daily range of color temperatures, and high CRI lighting (Light2). The high CRI lighting utilizes a light source from Company S, which promotes features of color temperature tuning and aims to replicate the spectrum of natural light. This selection allows for a comprehensive comparison of each lighting type’s effectiveness in color rendering performance. Considering the domestic certification standards for General Lighting, the distance between the light and the spectroradiometer was maintained at 150 cm when measuring artificial light. Figure 1 shows the results of measuring the spectrum of natural light. The key optical characteristic information related to color rendering derived from the measurement results is summarized in Table 1.

**Figure 2:**
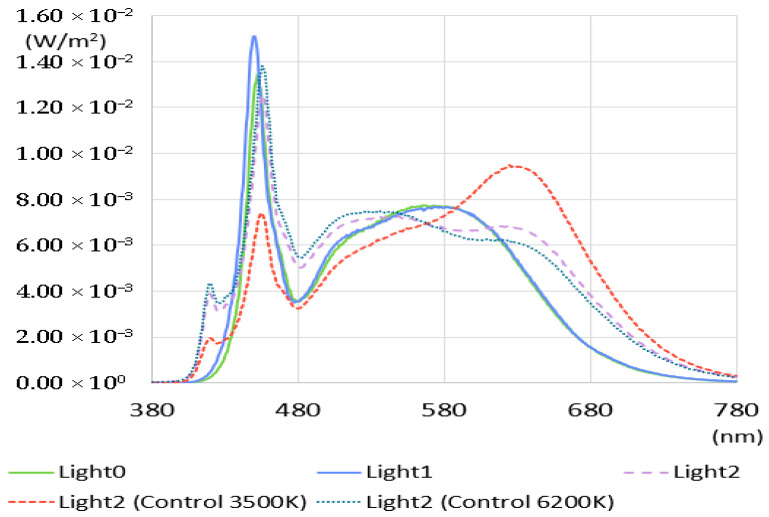
SPD of artificial light.

Figure 1 shows the spectral measurements at noon on a selected day for each season. The natural light spectrum on the selected days showed even distribution across all visible wavelength bands. Specifically, the intensity was slightly lower in the range below 430 nm and above 730 nm. Meanwhile, the color temperature at noon on each selected day was 5400–5700 K. The spectra at the minimum and maximum color temperatures during autumn days are displayed as dotted lines to present the characteristics of natural light according to changes in time and weather. At 6:59, when the color temperature was minimal around sunrise, the illuminance (18,845 Lux) and color temperature (3513 K) were low. The SPD showed an upward trend from the low wavelength band to the high wavelength band. At 10:23, when the color temperature was maximum, the illuminance was low at 26,042 Lux. Still, the color temperature was high at 6239 K. At this time, the difference in irradiance intensity by wavelength was not significant, displaying a flat spectral pattern, which might be due to the cloudy weather conditions. The CRI characteristics calculated for the measured SPDs in Figure 1 are summarized in Table 1. The SPD patterns of natural light varied depending on the season and time, but the color rendering performances were high in all SPD conditions. At 6:59 AM during the fall, the CRI, R9, and R12 values were at their lowest, measuring 96.1, 81.6, and 96.7, respectively. However, under different spectral power distribution (SPD) conditions, CRI, R9, and R12 values increased significantly, exceeding 99, 98, and 97, respectively. Therefore, during low-light conditions around sunrise or sunset, natural light consistently exhibited a CRI of 95, R9 of 80, and an R12 of 95 or higher, demonstrating exceptional color rendering performance. The CRI measurement results for artificial light are presented in Figure 2 and Table 2.

The spectrum of artificial light showed a different distribution from natural light, as shown in Figure 2. Artificial light, like natural light, could not contain a spectrum across the entire visible wavelength range and exhibited a high spectral pattern near a particular wavelength band (450 nm). The spectral intensity was weak, especially in the region below 430 nm. These characteristics were slightly more pronounced in Light0 and Light1, and both lights showed poor color rendering performances, with CRI below about 85 and R9 below 20. Light2, where a high CRI light source was applied, measured the spectrum (when controlled to 3500 K and 6212 K) similar to natural light’s lowest and highest color temperatures. Light2 showed a slightly higher spectral pattern in the wavelength range below 430 nm than other artificial lights. When the color temperature was controlled to 3500 K, the spectral pattern in the wavelength band near 630 nm was remarkably high. This analysis confirmed that artificial light has lower color rendering performance overall compared to sunlight. Even for lighting aiming for high CRI, it was not easy to provide color rendering performance at the level of natural light at R9 and R12.

**Figure 3:**
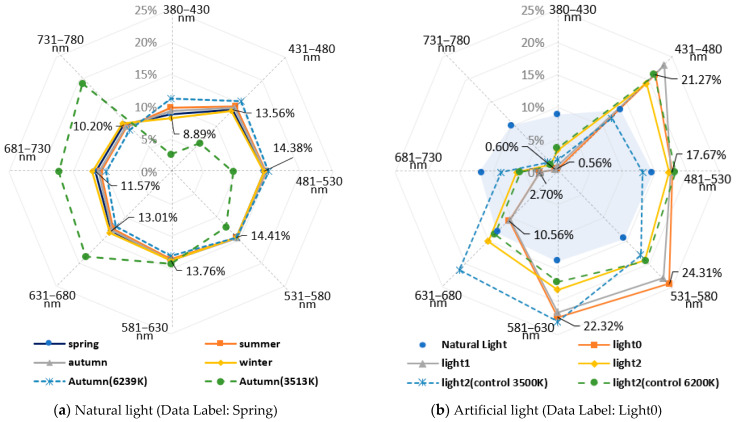
The spectral ratio of natural and artificial light. (**a**) Natural light (Data Label: Spring); (**b**) Artificial light (Data Label: Light0).

CRI is an evaluation index of color rendering performance with natural light as a reference. To achieve color rendering comparable to natural light, it is essential to replicate the spectrum of natural light perfectly, which is currently very challenging with existing technology. In this study, we calculated the ratio for each wavelength band to express how closely artificial light resembles natural light. Referring to previous research cases, the visible light wavelength band (380–780 nm) was divided into eight wavelength band sections at intervals of 50 nm [12,13]. Afterwards, the ratio for each wavelength section (Spectral Ratio, $R_{{SPD}_{N}}$) was calculated using Formula (1). The results are shown in Figure 3 and Table 3.

(1) $$R_{{SPD}_{N}}=\sum_{m}^{n}\lambda_{i}÷\;\sum_{380}^{780}\lambda_{i},\;\left(N=1,\ldots ,8\right)\;\;there\;is\;\left\{\begin{array}{c} N=1\;then\;m=380,\;n=430\\ N\;\neq 1\;then\;m=380+50*N+1,\;n=430+50*N \end{array}\right\}$$

As in Figure 3a, the spectrum of natural light varied due to the influence of season and weather; in the wavelength band of 380–780 nm, an even distribution ratio of approximately 9–15% was observed for each wavelength band. However, when the color temperature was low at 3513 K, the natural light spectrum was less in the 380–580 nm band and more in the 581–780 nm band. Despite these variations, natural light showed an even distribution ratio across all wavelength bands, although there were differences in height. The artificial light of Figure 3b showed a low spectral ratio of less than 0.6% in the wavelength band of 380–430 nm and less than 3% in the 681–780 nm band.

Even in the case of Light2, which claims a high CRI, the ratios in certain wavelength bands were either significantly low or significantly high, and the overall spectral ratio pattern was uneven, unlike natural light. The difference in the spectral distribution ratio also caused a difference in the color rendering performance of artificial and natural light.

## 3. Control Method of Natural Light LED Lighting to Achieve High CRI by Optimizing Spectral Ratio

### 3.1. Spectral Ratio Optimization as per Natural Light Characteristics

LED lighting can be adjusted by controlling the applied current to each channel, allowing for modulation of the spectral ratio for specific wavelength bands, and thus enabling control of optical characteristics [23,24]. This study proposes a method for optimizing the spectral ratio to improve the similarity to natural light and color reproducibility by controlling specific wavelength bands. Optimizing the spectral ratio improves color rendering performance by managing each light source that constitutes artificial light so that the wavelength ratio approaches that of natural light. A combination simulation of SPDs by wavelength band was performed; the spectral ratio was analyzed to derive an optimal control method for artificial light that can provide color rendering performance at the level of natural light.

**Figure 4:**
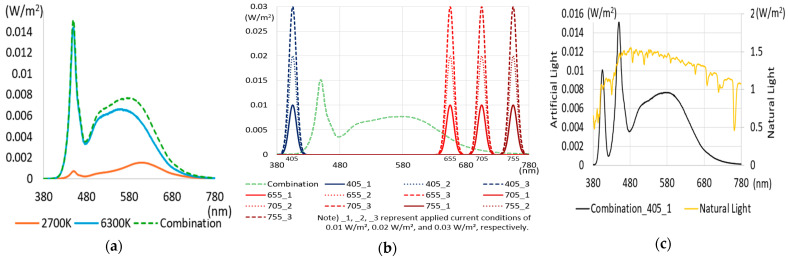
Spectral combination simulation. (**a**) SPD: LED light sources and Combination Light. (**b**) SPD: Combination Light + Virtual Light source. (**c**) Result of simulation.

To create artificial light close to natural light, selecting a light source is particularly important. It must provide high CRI performance while providing appropriate illuminances similar to existing general lighting. Popular commercial LED light sources form a spectral pattern with a high proportion of a specific wavelength band (around 450 nm) to increase luminous efficiency. However, this spectral pattern results in poor color rendering performance. Therefore, a combination of commercial LED light sources to secure illuminance and specific LED light sources to improve color rendering performance is required. For creating experimental lighting, two commercial LED light sources were first selected to provide sufficient illuminance and to control color temperature variation. If only two types of commercial LED light sources are used, achieving spectral ratio simulation of natural light and high color rendering performance is challenging. Therefore, a virtual light source was applied to compensate for the spectral deficiency in a specific wavelength band when combining artificial light sources. The virtual light sources have peak wavelengths of 405 nm, 655 nm, 705 nm, and 755 nm, respectively, and a FWMH of 25 nm. The intensity was simulated by adjusting it in three steps of 0.01 W/m^2^, 0.02 W/m^2^, and 0.03 W/m^2^. The simulation aimed to supplement the wavelength band lacking in artificial light and to explore the wavelength band most suitable for improving color rendering, such as R9 and R12. Figure 4 shows the simulation process of combining an LED light source and a virtual light source under a CCT condition of 5500 K after applying a general commercial LED light source with a CCT of 2700 K and 6500 K.

Figure 4a shows individual spectra for two commercial LED light sources (2700 K and 6300 K). The combined spectrum generated by adding the spectra of each light source is expressed as a dotted line. Figure 4b is a combination simulation calculation process that applies a virtual light source to the combination spectrum of Figure 4a and generates a spectrum for each applied current. The SPD of the virtual light source was configured in three stages, assuming the variable intensity of the applied current, so that the deficient wavelength band could be supplemented. Figure 4c The simulation result with the additional light source of 405 nm and the SPD of natural light under 5500 K CCT conditions is also shown. Examples of simulation results with the further application of a virtual light source are shown in Table 4.

As shown in Table 4, by applying additional light sources for each peak wavelength, the wavelength ratios of the 380–430 nm, 631–680 nm, 681–730 nm, and 731–780 nm bands were somewhere between 9.2 and 30.2%, which was higher than the combination light of the experimental lighting. In addition, the changes were observed in the light characteristics, such as illuminance, color temperature, and CRI. The color rendering performance changed significantly when an additional light source with a peak wavelength of 655 nm was applied. The color rendering performances of CRI, R9, and R12 all improved. However, the color temperature at this time was approximately 4500–5100 K, and the change in light characteristics due to the additional light sources was significant. Next, when an extra light source with a peak wavelength of 405 nm was applied, CRI, R9, and R12 increased slightly. In addition, when an additional light source with a peak wavelength of 705 nm and 755 nm was applied, the improvement in optical characteristics and color rendering performance was minimal. Other simulations, aside from the examples in Table 4, were also conducted. The color rendering performance simulations for color temperature conditions of 3500 K and 6200 K were performed and analyzed. The results showed that the wavelength ratio of the 380–430 nm and 631–680 nm bands needed improvement. When the wavelength ratio of the 631–680 nm band was controlled, the instability of the color temperature increased. Therefore, to enhance CRI, R9, and R12 and to maintain stable color temperature, a method for optimizing the wavelength ratio was derived by improving the wavelength band of 380–430 nm first and then supplementing the ratio of the wavelength band of 631–680 nm.

### 3.2. Control of High CRI Natural Light LED Lighting

**Figure 5:**
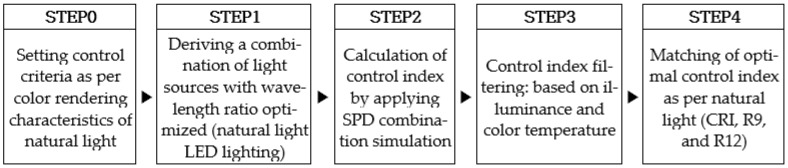
Optimal control method for natural light LED lighting.

To provide high color rendering performance at the level of natural light, (1) a control standard based on the characteristics of natural light, (2) a natural light LED lighting capable of providing this, and (3) a control method for LED lighting capable of providing a wavelength ratio similar to natural light are required. An optimal control method for natural light LED lighting that realizes these requirements and methods has been developed, and the process flow of the proposed method is shown in Figure 5.

**Figure 6:**
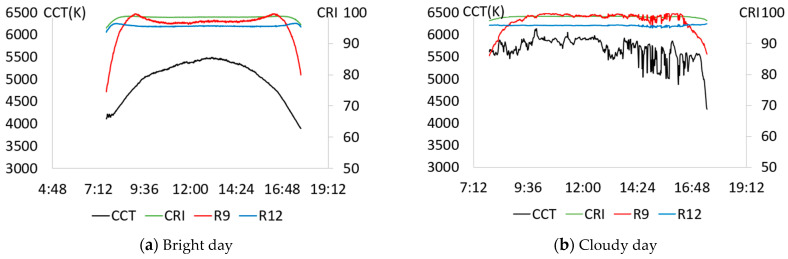
Daily color rendering performance changes in natural light. (**a**) Bright day; (**b**) Cloudy day.

First, as in STEP 0, we analyzed the characteristics of actual natural light to establish lighting control criteria based on the color rendering characteristics of natural light. After selecting one bright and one cloudy day, the hourly color temperature and color rendering characteristics (CRI, R9, and R12) of each day were analyzed. The results are shown in Figure 6.

Figure 6a shows the change in natural light color rendering performance on a bright day (10 March 2021). On bright days when the color temperature showed a stable change pattern in a parabolic shape, the CRI was remarkably high, reaching values of about 95 or higher and close to 100. R12 was a similar value, although slightly lower than CRI, and R9 was confirmed to be 75–100. The irregular flow of color-temperature of Figure 6b suggested that it was not a bright day. On cloudy day (7 March 2021) of Figure 6b, CRI and R12 were high at over 95, and R9 was over 85, showing even higher values than on bright days. Natural light provided light with a high color rendering index of CRI, R9, and R12 above 95 most of the time during the day, regardless of weather conditions. Considering these characteristics, the control standard for high CRI lighting was set as CRI and R12 to be higher than 95 and to provide a color rendering performance of the highest possible R9 even under various light and color conditions of natural light.

In STEP 1, we developed experimental lighting (natural light LED lighting) to optimize these spectral ratios. Based on the analysis results from Section 3.1, a light source that enhances the 380–430 nm wavelength band was requested from the lighting source developer. Four types of LED light sources suitable for experimental lighting were identified. Two types of WW and WC LED light sources were selected to provide diverse natural light colors while satisfying general domestic lighting standards’ recommended illuminations (300–600 Lux). The two light sources have color temperatures of 3000 K and 6500 K, respectively. They are commercial LED light sources (Allix Co., Ltd., Jeonju, Republic of Korea) with a CRI of 95 or higher that can provide high CRI due to the relatively high proportion of the 630–680 nm wavelength band. However, it is challenging to meet the spectral requirements of LED lighting’s deficiency band and produce a wide range of colors with only two light sources. Therefore, two light sources with peak wavelength characteristics of 405 nm (Allix, Jeonju, Republic of Korea) were additionally applied to improve the ratio of the 380–430 nm wavelength band. A total of 4CH individually controllable LED lightings were fabricated, one type each of WC and WW LED with a peak wavelength of 450 nm at CH1 and CH2, respectively, and one type each of WC and WW LED with a peak wavelength of 405 nm at CH3 and CH4. Table 5 presents details about the light sources used in the experiments, specifically natural light LEDs. The commercial light sources had a luminous efficacy (lm/W) ranging from 44 to 67, with an average CRI of over 97.

**Figure 7:**
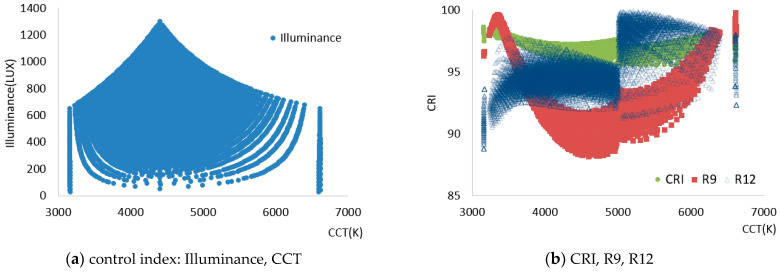
Results of applying simulation with SPD combination to derive control index. (**a**) control index: Illuminance, CCT; (**b**) CRI, R9, R12.

In STEP 2, the optical characteristics were collected according to the control of each channel/applied current to provide optimal color rendering performance even under the color conditions of natural light that change colorfully throughout the day, using natural light LED lighting with the four light sources as in Table 5. A spectral-based calculation was performed to control the input current of each channel of natural light LED lighting, which increased by 10% stepwise. The resulting 14,641 optical characteristics were derived according to the combination control for each channel, and the results are shown in Figure 7.

The controllable range of natural light LED lighting can also be confirmed in Figure 7. As shown in Figure 7a, providing 3200–6700 K CCT within a maximum illuminance of 1200 Lux was possible. In addition, as in Figure 7b, natural light LED lighting showed a CRI of over 95 in all color temperature conditions and an R12 distribution of over 90. However, R9 showed a value of about 88 to 100, displaying a significant variation. As shown in Table 5 and Figure 7, the combination of selected light sources effectively supplements the insufficient wavelength band of existing artificial lighting and addresses the deficiencies in the R9 and R12 characteristics.

In STEP 3, a control index was established to achieve an illuminance level between 300 and 600 Lux, based on the recommended domestic standards for general lighting, as illustrated in Figure 7a. Afterward, the control indices that could give the hourly color temperature of natural light within the range of ±50 K were filtered from Figure 7b. In STEP 4, after filtering the control indices based on illuminance and color temperature, the optimal control indices per the color rendering properties of natural light were matched. First, the control indices with a CRI of 95 or higher were extracted. Then, control index candidates with an R12 of 95 or higher were extracted. Among the candidates for the control indices to control LED lighting, the control indices that could provide the highest R9 were selected. Applying the control indices extracted through this series of optimal matching processes controlled the high CRI natural light LED lighting, thereby providing color rendering performance at the level of natural light.

## 4. Experiments and Discussion

**Figure 8:**
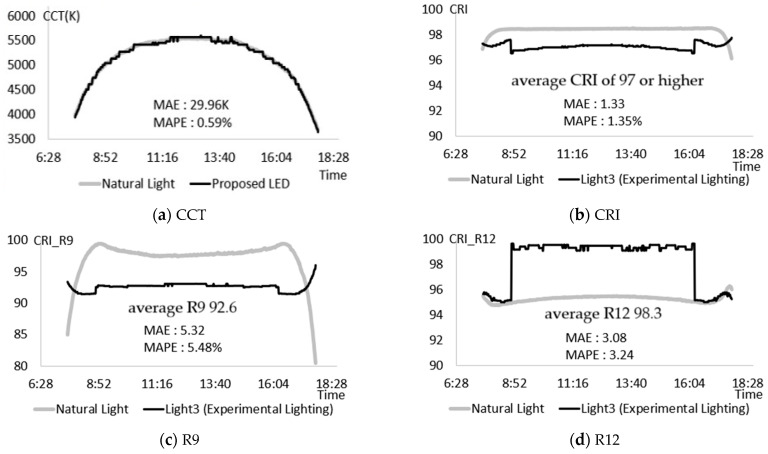
Experiment to reproduce the color rendering performance of natural light. (**a**) CCT; (**b**) CRI; (**c**) R9; (**d**) R12.

To evaluate the performance of the proposed method, experiments were conducted from two perspectives: how realistically the high CRI natural light LED lighting (Light3) fabricated in this study can reproduce the color rendering performance of actual natural light and whether the proposed method can be applied to the development of commercial LED lighting. First, under the changing CCT conditions of a bright day (10 March 2021), the control criteria for high CRI natural light LED lighting that provided light of CRI and R12 of 95 or higher and R9 of 90 or higher were set. Control criteria were set at one-minute intervals, totaling 587 measurements, ranging from the lowest point of CCT after sunrise (07:35) to the lowest point of CCT before sunset (17:49). After optimizing the control index to meet these criteria, the settings were applied to each channel of high-color-rendering natural light LED lighting. The results are depicted in Figure 8.

**Figure 9:**
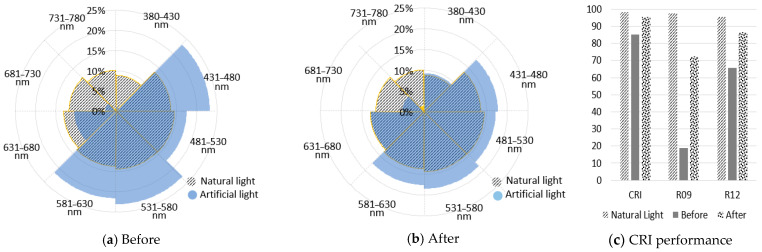
Results of applying Light1, proposed method. (**a**) Before; (**b**) After; (**c**) CRI performance.

As shown in Figure 8a, the color temperature of colorful natural light was successfully reproduced within a range of approximately 3500 to 5500 K, with a Mean Absolute Percentage Error (MAPE) of 0.59%. Figure 8b demonstrates that the CRI achieved a performance MAPE of 1.35%, providing an average CRI of 97 or higher. Additionally, R9 in Figure 8c exhibited a high average value of 92.6, although its MAPE was 5.48%, attributed to the notable variation between sunrise and sunset. Finally, R12 in Figure 8d revealed an impressive average CRI of 98.3 with a MAPE of 3.24%. Therefore, the proposed method could provide color reproduction performance similar to natural light under continuously changing color temperature conditions. After that, the general applicability of the proposed method was analyzed through experiments on two types of lighting used to analyze the characteristics of artificial light. Two types of 405 nm peak light sources were applied to Light1 (CCT Tunable Lighting) with color temperature control, and Light2 with a color temperature control function, as well as a high CRI light source. Figure 9 shows an example of using the proposed method on Light1 under a color temperature condition of 5500 K.

As shown in Figure 9a, there was a spectral deficiency in the wavelength range of 380 to 430 nm, but the spectral ratio of the range was supplemented through the additional application and control of the 405 nm peak light source. The results showed that, as in Figure 9b, not only was the ratio of the wavelength range of 380 to 430 nm enhanced, but the remaining ranges were also adjusted to be closer to the spectral ratio of the wavelength band of natural light. Figure 9c shows the result of comparing the color rendering performance. CRI increased by about 10% from before the application to 95, and R9 rose from 18.69 to 72.25 after applying the proposed method. Furthermore, R12 significantly improved from 65.71 to 86.58. The results of applying the proposed method to all experimental lights, having classified the natural light LED lights as Light3, are presented in Table 6.

As shown in Table 6, the color reproduction performance of Light1, Light2, and Light3 was analyzed under color temperature conditions of 3500 K, 5500 K, and 6200 K, considering the light color of natural light. Light1 was able to closely replicate natural light in terms of CRI and R12 values, with R9 also improving significantly to over 70 after application. Light2 and Light3 maintained a 3 to 9% spectral ratio in the 380 to 430 nm wavelength band, similar to natural light. For Light2, CRI was over 96, and R9 and R12 showed values of about 95 and 91 at color temperatures of 3500 K and 5500 K, respectively. In addition, for the Light3, under color temperature conditions of 6200 K, cloudy conditions, or around sunrise and sunset, R9 was over 94, and R12 was about 90. Meanwhile, under color temperatures of 3500 K and 5500 K, the color of general natural light, CRI, was over 97, R9 was about 93, and R12 was about 95, providing color rendering performance close to natural light. When the TM-30 indicator, which is commonly used to assess color rendering performance, was applied, the results showed high values. Specifically, at a correlated color temperature (CCT) of 3500 K, the values were Rf = 97.576 and Rg = 100.607. At 5500 K, the values were Rf = 97.247 and Rg = 102.611, and at 6200 K, they were Rf = 97.593 and Rg = 101.718. Through experimental lighting, a light was produced that exhibited high color rendering performance comparable to that of natural light for most of the day. This confirms that the proposed method can markedly enhance the color rendering performance of general LED lighting.

## 5. Conclusions

Despite efforts to achieve high color rendering performance by simulating natural light, a generalized technique for reproducing spectral characteristics could not be established. This study proposes an optimized control method based on the spectral ratio characteristics of natural light LED lighting, successfully achieving high color rendering. First, the actual spectral ratio characteristics were compared and analyzed to confirm the deficiency wavelength band (380–430 nm, 680 nm or more) of artificial light that was insufficient compared to natural light. It was determined that the presence of the deficient wavelength band caused a deterioration in color rendering performance. A simulation was performed using a virtual light source to reinforce this limitation. Assuming general lighting, a spectrum for a CCT condition of 5,500 K was generated using commercial LED light sources of 2700 K and 6500 K, and then the virtual light source was combined. Finally, the spectral wavelength ratio optimization method that sequentially reinforced the 380–430 nm and 631–680 nm wavelength bands was derived. Afterward, two types of commercial light sources with CRI ≥ 95, which have a high ratio of wavelength bands over 630 nm, and two other types of light sources with peak wavelength characteristics of 405 nm for reinforcing the 380–430 nm wavelength band, were applied to fabricate 4CH natural light LED lighting. In addition, a method for controlling natural light LED lighting that provides color temperature by time was developed through channel-by-channel control of LED lighting while matching CRI ≥ 95, R9, and R12 to be close to natural light. For performance evaluation, the natural light of a bright day was selected as the standard, and the proposed method was applied to natural light LED lighting (Light3). The results showed an average CRI of 97, with R9 scoring around 93 and R12 reaching 90 or higher under varying color temperature conditions of 3500 to 6200 K, demonstrating color rendering performance close to that of natural light. Additionally, when experimenting with the proposed method, the color temperature-variable lighting (Light1) featuring a typical LED light source recorded CRI and R12 values of over 97 and 92.6, respectively, marking a substantial enhancement in CRI compared to existing lighting solutions. The high CRI lighting (Light2), which uses a high color rendering source, also exhibited improved R9 and R12 values of 95 or higher and 91 or higher, respectively, under color temperature conditions of 3500 K and 5500 K. Therefore, the proposed method can effectively improve the color rendering performance of general LED lighting.

In the future, we plan to conduct research to improve the performance of the proposed method, such as by additionally applying a light source with a specific peak wavelength (e.g., 630 nm) to reproduce the wavelength ratio of natural light in more detail. In addition, when developing a commercial lighting system based on the proposed method, further experiments are needed to assess overall lighting efficiency. Efforts will be made to create a lighting system that supports linked control and verification of light quality in response to changes in natural light, such as weather conditions.

## Figures and Tables

**Table 1:** Optical characteristics of natural light.

Light Source	Illuminance (Lux)	CCT (K)	CRI		
			(Ra)	(R9)	(R12)
Spring (17 March 2021)	97,667	5471	99.3	98.8	97.8
Summer (3 June 2023)	113,055	5742	99.2	99.3	97.4
Autumn (27 September 2022)	113,729	5607	99.3	98.1	97.8
Winter (24 December 2024)	107,135	5402	99.2	99.6	97.6
Autumn (27 September 2022. 10:23)	26,042	6239	99.3	99.0	97.8
Autumn (27 September 2022. 06:59)	18,845	3513	96.1	81.6	96.7

**Table 2:** Optical characteristics of artificial light.

Light Source	CCT (K)	CRI		
		(Ra)	(R9)	(R12)
Light0 (General Office Lighting)	5200	82.7	4.12	59.6
Light1 (CCT Tunable Lighting)	5516	85.3	18.6	65.7
Light2 (High CRI Lighting)	5513	97.5	93.2	82.7
Light2 (Control: 3500 K)	3500	97.4	94.4	88.8
Light2 (Control: 6200 K)	6212	96.0	85.8	79.0

**Table 3:** Details of spectral ratio of natural and artificial light.

Category Wavelength (nm)	Spectral Ratio (%)										
	Natural Light						Artificial Light				
	Spring	Summer	Autumn	Winter	Autumn (6239 K)	Autumn (3513 K)	Light0	Light1	Light2	Light2 (Control 3500 K)	Light2 (Control 6200 K)
380–430	8.89	9.90	9.34	8.25	11.26	2.64	0.56	0.94	3.37	1.87	3.72
431–480	13.56	14.24	13.91	13.22	15.36	6.27	21.27	23.21	19.31	11.75	21.12
481–530	14.38	14.67	14.62	14.39	15.11	9.68	17.67	17.47	17.18	13.11	18.15
531–580	14.41	14.42	14.49	14.31	14.31	12.08	24.31	23.06	19.10	18.08	19.34
581–630	13.76	13.51	13.67	13.70	12.99	14.24	22.32	21.58	18.09	23.04	16.91
631–680	13.01	12.58	12.81	13.34	11.92	18.54	10.56	10.39	15.07	21.34	13.57
681–730	11.57	10.82	11.10	12.09	10.03	17.31	2.70	2.72	6.28	8.67	5.71
731–780	10.20	9.65	9.85	10.49	9.02	19.25	0.60	0.61	1.59	2.12	1.46

**Table 4:** Results of spectral combination simulation (CCT of experimental lighting: 5500 K).

Light Source	SPD Current Value STEP	Spectral Characteristics (Spectral Ratio %)								Light Property				
		380–430 nm	431–480 nm	481–530 nm	531–580 nm	581–630 nm	631–680 nm	681–730 nm	731–780 nm	Illumin-ance (Lux)	CCT (K)	CRI	R9	R12
Natural Light		8.50	13.70	14.60	14.60	13.50	13.10	11.50	10.30	-	5500.0	97.2	91.5	82.8
Combination Light		1.1	23.4	17.9	23.5	21.9	10.5	2.7	0.6	499.2	5518.8	85.3	18.7	65.7
Add Light Source 405 nm	1	9.6	21.4	16.4	21.5	20.0	9.6	2.5	0.6	499.3	5607.3	85.7	21.1	68.8
	2	16.7	19.7	15.1	19.8	18.5	8.8	2.3	0.5	499.4	5699.7	86.0	23.6	71.6
	3	22.8	18.3	14.0	18.4	17.1	8.2	2.1	0.5	499.5	5796.2	86.3	26.0	74.1
Add Light Source 655 nm	1	1.0	21.4	16.4	21.5	20.0	18.2	2.5	0.6	508.0	5157.0	91.5	74.3	72.5
	2	0.9	19.7	15.1	19.8	18.5	24.6	2.3	0.5	516.8	4823.6	94.1	77.7	71.6
	3	0.8	18.3	14.0	18.4	17.1	30.2	2.1	0.5	525.6	4516.6	90.2	37.8	74.1
Add Light Source 705 nm	1	1.0	21.4	16.4	21.5	20.0	9.6	11.1	0.6	499.5	5504.1	85.6	21.5	65.6
	2	0.9	19.7	15.1	19.8	18.5	8.8	18.1	0.5	499.9	5489.4	85.9	24.2	65.6
	3	0.8	18.3	14.0	18.4	17.1	8.2	24.1	0.5	500.2	5474.7	86.2	26.9	65.5
Add Light Source 755 nm	1	1.0	21.4	16.4	21.5	20.0	9.6	2.5	9.2	499.2	5518.4	85.3	18.8	65.6
	2	0.9	19.7	15.1	19.8	18.5	8.8	2.3	16.3	499.2	5517.9	85.3	18.9	65.6
	3	0.8	18.3	14.0	18.4	17.1	8.2	2.1	22.5	499.2	5517.5	85.3	18.9	65.5

**Table 5:** Characteristics of light sources of experimental lighting by channel.

	CH1	CH2	CH3	CH4
Category	Peak: 450 nm, Illuminance: 400 Lux		Peak: 405 nm, Illuminance: 250 Lux	
SPD	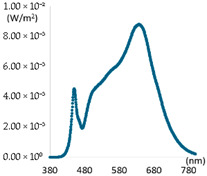	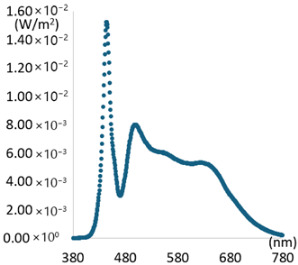	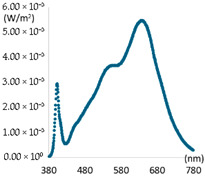	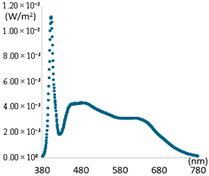
CCT(K)	3158 K	6621 K	3165 K	6601 K
CRI	98.61	96.34	97.44	95.89
R9	96.26	98.02	96.60	97.45
R12	88.80	92.32	93.58	93.85
lm/W	62	67	45	44

**Table 6:** Lighting control results after spectral ratio optimization.

CCT (K)	Light Source	Spectral Characteristics (Spectral Ratio %)								Light Property				
		380–430 nm	431–480 nm	481–530 nm	531–580 nm	581–630 nm	631–680 nm	681–730 nm	731–780 nm	Illumin-ance	CCT	CRI	R9	R12
3500	Natural Light	3.06	6.89	10.05	11.97	13.83	17.62	17.97	18.18	-	3687	96.1	80.4	96.0
	Light1 (CCT Tunable)	7.73	10.62	13.61	17.52	22.86	18.30	7.33	2.00	509.0	3547	96.0	74.0	94.3
	Light2 (High CRI)	3.08	10.94	13.13	17.83	22.65	21.26	8.85	2.24	494.1	3495	97.8	94.9	91.3
	Light3 (Experimental)	3.65	8.99	13.76	17.47	21.26	21.83	10.15	2.86	479.1	3502	98.0	97.6	95.1
5500	Natural Light	8.85	13.57	14.46	14.35	13.67	13.04	11.70	10.15	-	5500	98.4	97.8	95.4
	Light1 (CCT Tunable)	9.34	17.72	17.17	18.47	17.56	12.86	5.34	1.51	509.3	5493	95.4	72.3	86.6
	Light2 (High CRI)	9.96	16.46	17.13	17.17	16.54	14.41	6.49	1.82	509.3	5658	98.8	98.7	93.6
	Light3 (Experimental)	7.19	16.78	18.42	17.07	16.77	15.01	6.81	1.93	511.3	5481	97.1	92.8	99.1
6200	Natural Light	9.04	13.70	14.50	14.34	13.63	12.94	11.61	10.03	-	5547	98.4	97.6	95.5
	Light1 (CCT Tunable)	9.02	20.46	18.13	19.24	17.15	10.76	4.09	1.13	504.3	6178	92.2	53.2	80.0
	Light2 (High CRI)	9.87	18.57	18.01	17.43	15.80	13.00	5.73	1.59	499.9	6389	96.3	87.3	82.0
	Light3 (Experimental)	8.66	18.51	19.41	16.81	15.64	13.29	5.97	1.70	504.4	6155	98.8	94.1	89.8

## Data Availability

The datasets presented in this article are not readily available because the data are part of an ongoing study and due to technical/time limitations.
